# Supplementary material for: Biosynthesized Silver Nanoparticles for Cancer Therapy and In Vivo Bioimaging
Source: Cancers (Basel). 2021 Dec 4;13(23):6114. doi: 10.3390/cancers13236114 (PMC8657022; doi:10.3390/cancers13236114)
Supplement: Supplementary file 1 [file cancers-13-06114-s001.zip › cancers-1460624-supplementary.pdf]

Supplementary Materials: Biosynthesized Silver Nanoparticles for Cancer Therapy and In Vivo Bioimaging

Shagufta Haque, Caroline Celine Norbert, Rajarshi Acharyya, Sudip Mukherjee, Muralidharan Kathirvel and Chitta Ranjan Patra

2.2. Characterization

The characterization of AgZE is performed by different analytical techniques (DLS, XRD, FTIR, SEM, TEM, fluorescence, ICPOES) according to earlier published literature [1–3].

2.2.1. Dynamic light scattering (DLS)

The hydrodynamic diameter and the surface charge of the AgZE nanoparticles is determined by the dynamic light scattering (DLS) method using Litesizer500 particle analyzer (Anton Par) as per published literature [2]. The AgZE sample (50 μL)is diluted with 950 μL of water in a disposable plastic cuvette for size measurements where for each sample the instrument underwent 20 cycles. The charge of the nanoparticle (zeta potential) is measured using a charge omega cuvette following the same dilution as for size measurement.

2.2.2. X-ray diffraction (XRD) analysis

X-ray diffraction (XRD) method analyzes the surface crystallinity of the AgZE as per published literature [2]. The pellet of AgZE solution collected after centrifugation at 30,000 rpm is coated on a glass slide repeatedly until a thin layer is formedfor XRD analysis using Bruker AXS D8 Advance Powder X-ray diffractometer (using CuK$\alpha\lambda$=1.5406 A radiation) at 2θ = 20º to 80º].

2.2.3. Fourier transform infrared spectroscopy (FTIR) analysis

Fourier transform infrared spectroscopy (FTIR) analysis is a method that is carried out to analyze the presence of functional groups in the AgZE formation [3]. The AgZEpellet and ZE extract are dissolved in 100μl water individually for FTIR analysis (Thermo Nicolet Nexus 670 FTIR spectrometer). The FTIR analysis is performed with a resolution of 4 cm^{-1} in KBr pellets from a range of 400–4000 cm^{-1} in the diffuse reflectance mode.

2.2.4. Scanning electron microscopy (SEM)

The scanning electron microscopy (SEM) analysis analyzes the surface morphology of AgZE. Theyellowish green coloured liquid pellet obtained after centrifugation is used for SEM using SEM Hitachi S-3000N, Japan.

2.2.5. Transmission electron microscopy (TEM)

The shape, size and morphology of the AgZE are analyzed by the transmission electron microscopy using TEM: Tecnai G2 F30 S-Twin Microscope operated at 100 kV [3]. The yellowish green coloured liquid pellet obtained after centrifugation is coated onto a carbon coated copper grid and allowed to dry. The dried sample is then analysed by TEM.

2.2.6. Fluorescence measurements

The fluorescence activity of the AgZE extract is evaluated using multimode fluorescence reader (Synergy H1). Initially, the AgZE pellet is taken in a 96-well black plate (100 μL volume) followed by series range of excitation starting at 350 nm and emission starting at 450 nm. The resulting fluorescence emission spectrum is recorded and expressed in the graphical form of RFU (relative fluorescence unit).

2.3. In vitro experiments

2.3.2. Cell viability assay using MTT reagent

MTT is a cell viability assay performed to assess the effect of AgZE nanoparticles on the normal cell lines (CHO, HEK-293T, EA.hy926 and H9c2) for two time points (24 h and 48 h) and cancer cell lines (MCF-7, U-87, PANC-1, HeLa and B16F10) for 48 h as per earlier published protocol [3]. The experiments are performed in triplicates. The normal and cancer cells are seeded into 96-well plate (8000 cells/well), grown to confluence and treated with AgZE in a dose dependent manner (1-10 μL). After the respective time points of treatment, the culture media is replaced with MTT reagent (0.05 mg/mL) in each well and incubated for 4 h under dark conditions. Then a mixture of DMSO/methanol (1:1 ratio) is added to each well (100 μL) replacing the MTT reagent to solubilize the *in situ* formed formazan crystals. The intensity of the color produced is measured at 570 nm using a Synergy H1 multimode plate reader system. The viability of the cells is expressed as normalized values with respect to control untreated cells.

2.3.5. Cell cycle assay

The effect of AgZE on the cell cycle progression for cancer cell line (U-87) is carried out using propidium iodide staining as per published literature [2]. Temozolomide is used as positive control. U-87 cells are cultured in 60 mm dishes, treated with the AgZE and incubated for 24 hours. The cells are then washed with DPBS buffer two times, centrifuged and pellet is fixed with 70% ethanol for storage in -20°C overnight. After incubation, the samples are again centrifuged at 3000 rpm for 1-2 min followed by addition of PI mix and incubating at 37°C for about 30–45 min. Later, the solution is centrifuged at 3000 rpm for 2 min and the pellet is dissolved in 500 μL of DPBS buffer for analysis using FACS Canto II, Becton Dickinson, San Jose, CA, U.S. The data are examined using F3 express software.

2.3.6. Cellular apoptosis assay

The apoptotic effect of AgZE on U-87 cancer cells is analyzed by cell apoptosis assay by Annexin-V and propidium iodide stain in flow cytometry [2]. The cells are cultured in 60 mm dishes for 24 h, treated with AgZE and temozolomide (positive control) followed by incubation till 24 h. The same procedure is repeated for 30 h. The cells are then trypsinized and washed with DPBS. The supernatant is discarded and the pellet is processed with Annexin V- FITC staining kit as per manufacturer's protocol. The labeled cells are analyzed in FACS Canto II, Becton Dickinson, San Jose, CA, U.S.

2.3.7. Reactive oxygen species (ROS) assay

ROS generation of AgZE on the U-87 cells is carried out as per earlier published protocol [4,5]. The experiments are performed in triplicates. Initially, U-87 cells (25,000 cells/well) are cultured in 24 well plates, treated with AgZE, incubated for 5 h under aseptic conditions. Tertiary-Butyl hydroperoxide (TBHP) is used as positive controlwhere it is given (100 μM) in the designated wells and incubated for 1 h. The same experiment is also performed for 24 h. Cells are then washed with DPBS and incubated with fluorescent probes 2′,7′ −dichlorofluorescin diacetate (DCFDA, for hydrogen peroxide: 10 mM) under dark conditions for 37 min. Cells are washed with DPBS and checked under a fluorescence microscope (Nikon Eclipse TE2000-E) at green fluorescence emission

(λ_{Em} = 535 nm) range since excitation at λ_{Ex} = 420–495 nm is for hydrogen peroxide and recorded with a 20× microscope objective.

2.3.8. Western blot analysis

The western blot analysis is performed as per previously published protocol [3]. The U-87 cells are seeded in a 60 mm dish, cultured till 80% confluency, treated with AgZE and temozolomide (positive control) followed by incubation of 18 h. The protein samples are prepared in RIPA buffer (Radio immunoprecipitation assay buffer) containing protease inhibitor cocktail (PIC) in 1000:10 ratio. The samples are centrifuged at 12,000 rpm/ 4°C/ 20 min and the clear supernatant is collected. The total concentration of proteins in the supernatant is calculated by Bradford assay. Then 50 μg protein of all samples (equal concentration) are separated on a 10% SDS-PAGE which is then transferred to a poly (vinylidene difluoride) [PVDF] membrane (Merck Millipore). Nonspecific sites on the proteins are blocked using 3% BSA for 1 h 30 min and washed thrice with 1× TBST (tris buffered saline with Tween 20). The membrane is then incubated with the respective primary antibodies, anti-caspase-8, anti-p53, anti-STAT3 and anti-GAPDH (as protein loading control) where the dilutions are done in accordance with the manufacturer's instructions for overnight at 4°C. The membrane is further washed with 1× TBST solution three times followed by incubation with goat anti-rabbit/mouse IgG HRP antibodies at room temperature for 1 h. The immunoblot is developed by chemiluminescence instrument (Fusion solo S, VilberLourmat) using Super signal west pico plus chemiluminescent substrate (Thermo Scientific).

2.4. In vivo assays

2.4.1. Chorioallantoic membrane (CAM) assay

Chorioallantoic membrane (CAM) assay is used to analyze the effect of the AgZE on the blood vessels over the chorioallantoic membrane in development stages of chick as per our earlier published reports [1]. Fertilized eggs are collected from Directorate of Poultry Research, Mehdipatnam, Hyderabad. The eggs are then kept in the incubator at 98.7° F and 65% relative humidity for 4 days. After 4 days a small crack is made at the bottom of the egg and around 3ml of the albumin is taken out and discarded. The hole is sealed with cello tape. The shell is then slowly cracked at the top which is then removed and checked for the formation of blood vessels. The eggs are then treated with the AgZE nanoparticles and images are taken at different time points (at 0 and 4 h) for comparison with untreated control eggs using Leica Stereo Microscope. The changes in blood vessels with reference to length, size and junctions are analyzed using Angio-Quant software.

Table S1. Synthesis, optimization and hydrodynamic diameter of AgZE with different reaction conditions.

Entry No	ZE extract(μL)*	$AgNO_3$ (μL)	Water (mL)	Hydrodynamic diameter(nm)
1	100	250	4.650	104.0
2	200	250	4.6	84.3
3	300	250	4.450	76.5
**4**	**500**	**250**	**4.25**	**95.7**

*[1:1 dilution with ethanol]; The total volume of each reaction is 5 Ml.

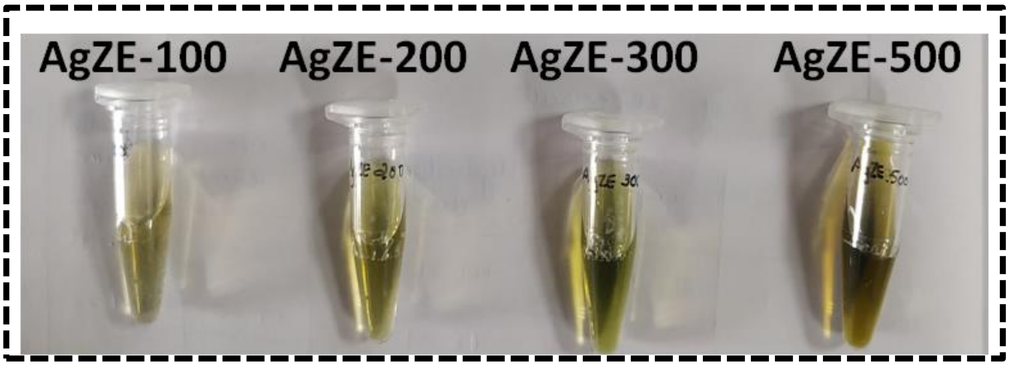

Figure S1. The representative picture of different AgZE reaction sets with reference to Table S1. The AgZE-500 (entry no #4) is the optimized reaction.

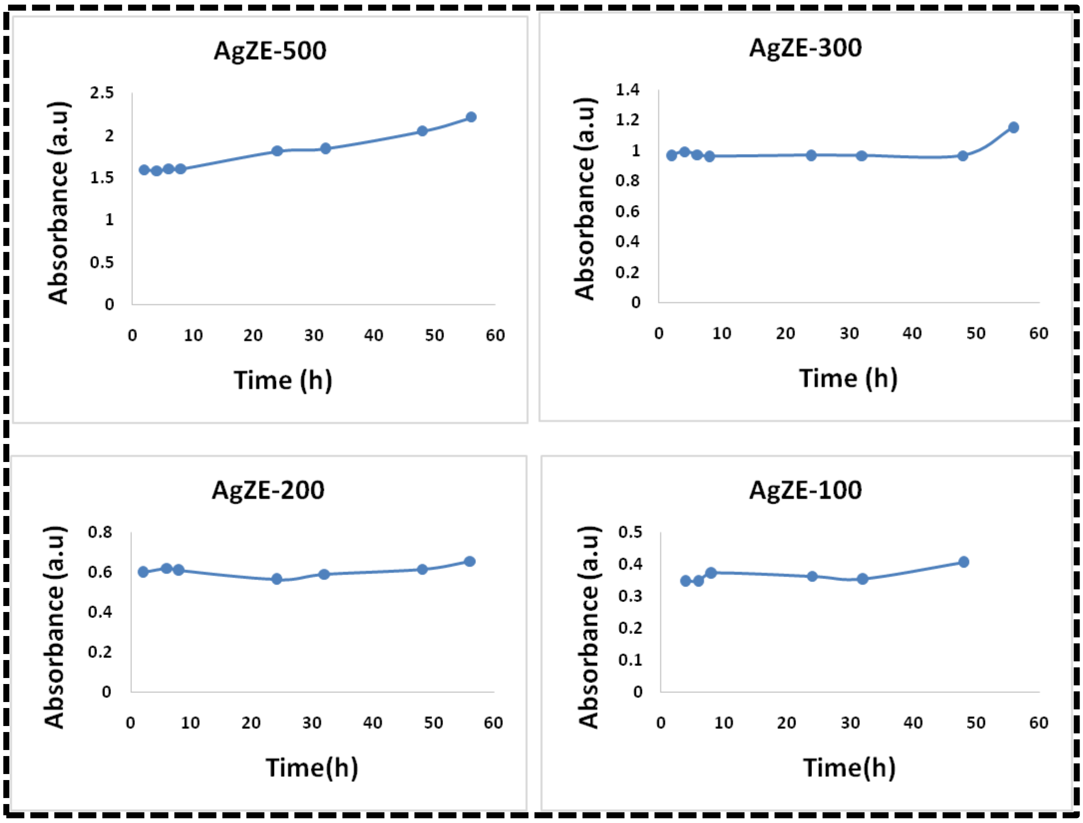

Figure S2. The UV absorbance spectra of different AgZE reaction sets at time points from 0-60 h. The AgZE-500 is the optimized reaction showing stable absorbance at 420 nm which is the characteristic peak for silver nanoparticles.

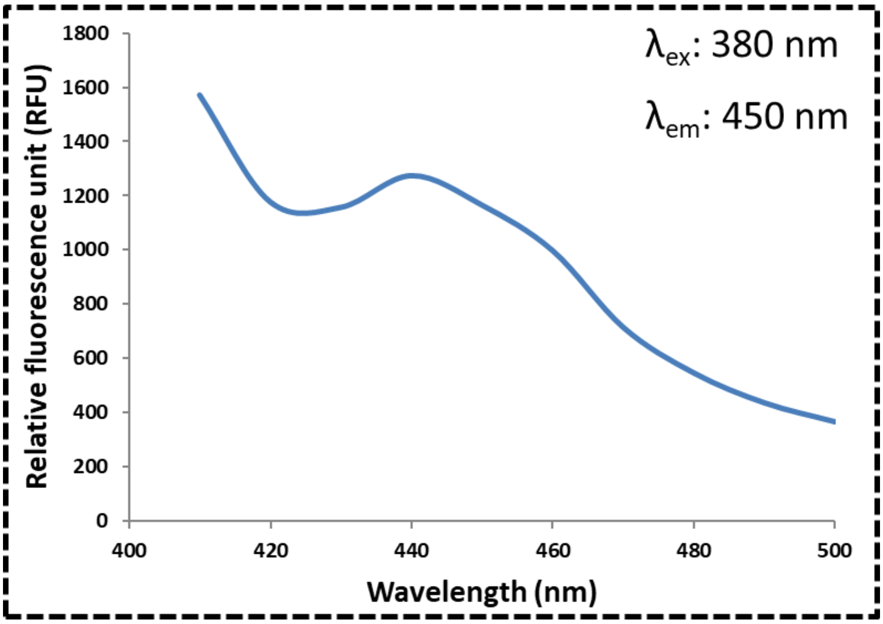

Figure S3. The fluorescence absorption of the AgZEat 380 nm excitation and 450 nm emission.

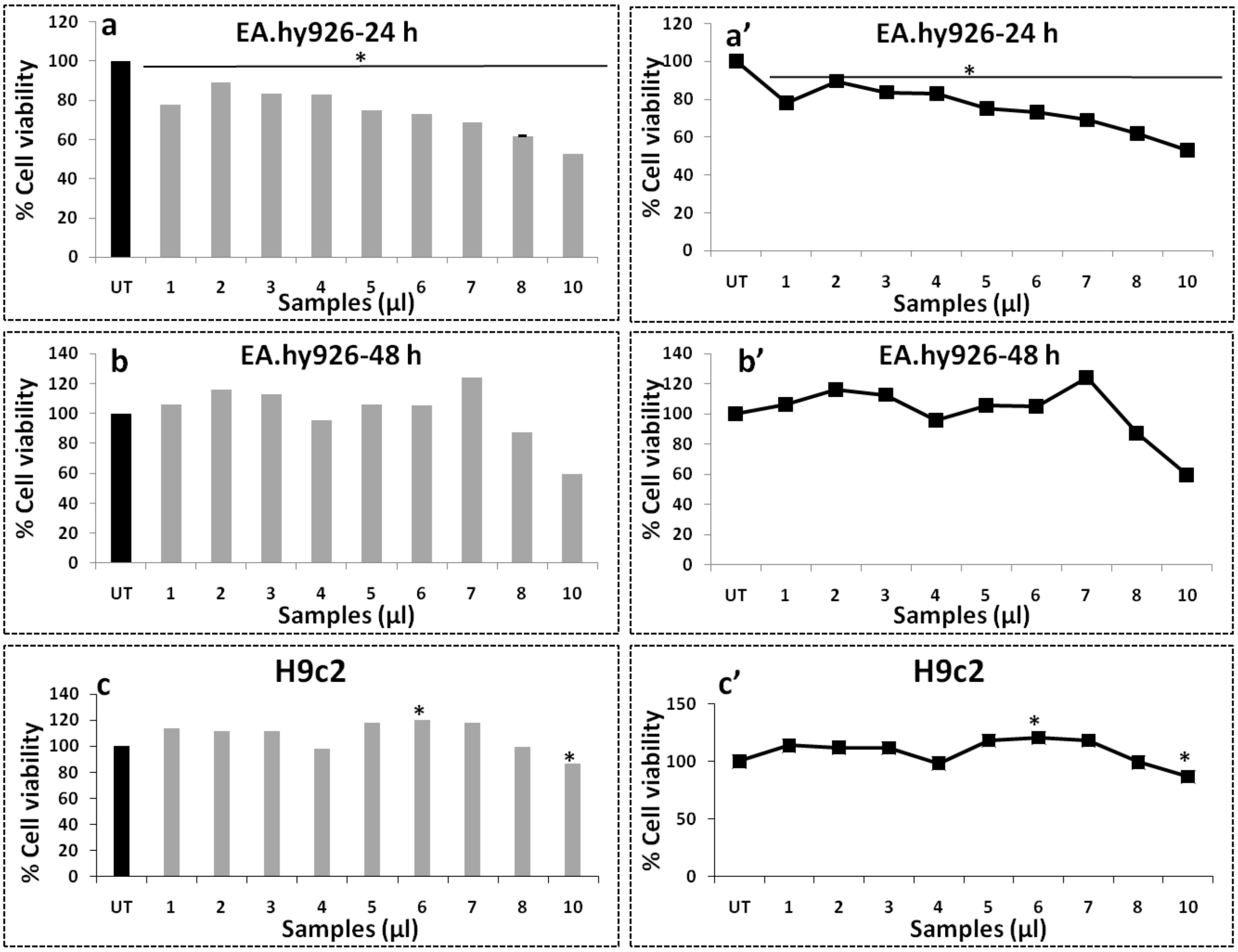

Figure S4. Cell viability analysis of AgZE using endothelial cell line (EA.hy926) by MTT. The dose-dependent cell viability study (using MTT reagents) of **a)** EA.hy926 (24 h) **b)** EA.hy926 (48 h) **c)** H9c2

(48 h) cells incubated with AgZE indicating biocompatibility at the therapeutic dose of around 5 μl/ml w.r.t. cancer cells. The corresponding dose response curves of **a′)** EA.hy926 (24 h) **b′)** EA.hy926 (48 h) **c′)** H9c2 (48 h) cells. (Numerical values indicate the AgZE pellet amount taken where 1μl corresponds to 0.23 μg/ml of silver as per ICPOES study). TEMO corresponds to temozolomide as positive control. Significant differences from untreated (UT) cells are observed (*$p < 0.05$).

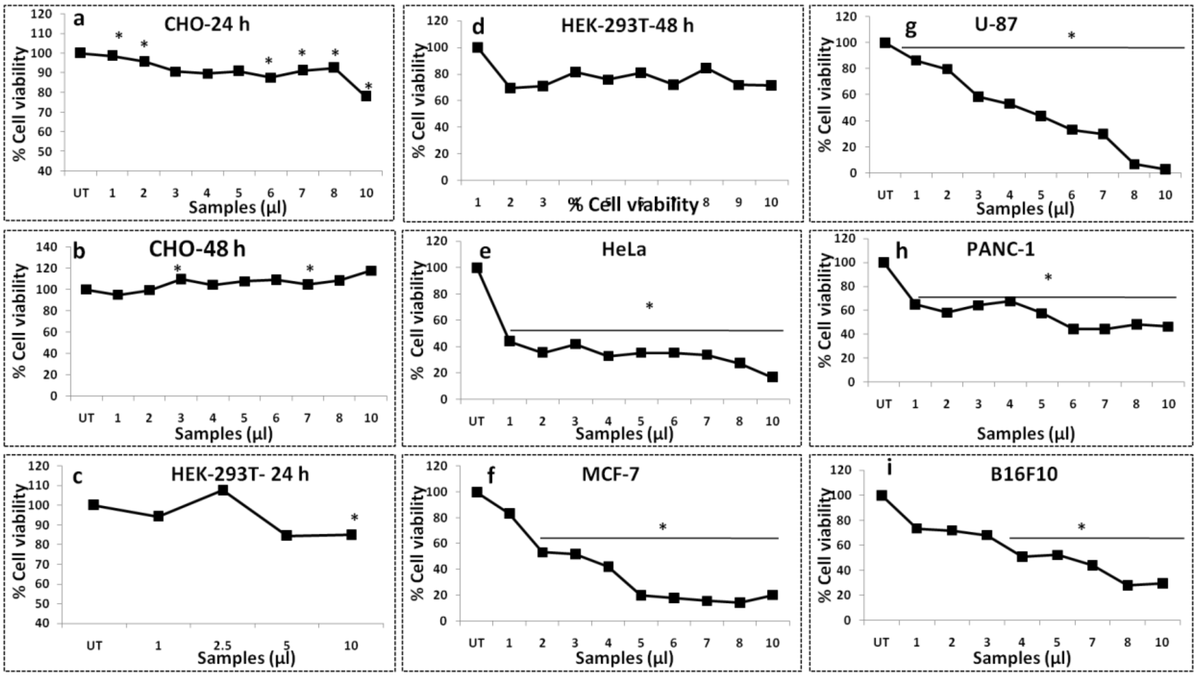

Figure S5. Cell viability of AgZE in normal and cancer cell line using MTT in the form of dose response curves. The cell viability assay of different normal (24 h and 48 h) and cancer cell lines (48 h) incubated with AgZE using MTT reagent in the form of dose dependent curves. The normal cell lines are: **a)** CHO (24 h), **b)** CHO (48 h), **c)** HEK-293T (24 h), and **d)** HEK-293T (48 h); the cancer cell lines are: **e)** HeLa **f)** MCF-7 **g)** U-87 **h)** PANC-1 **i)** B16F10. There is dose-dependent increase in cytotoxicity of AgZE towards cancer cells compared to normal cells indicating their anticancer activity (Numerical values shows the amount of AgZE taken where 1mL = 0.23 μg/mL of silver as per ICPOES study). Significant differences from untreated (UT) cells were observed (*$p < 0.05$).

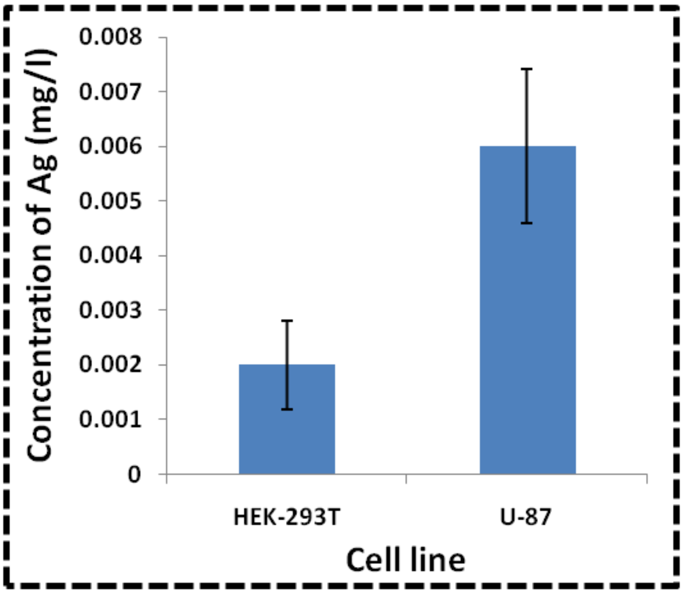

Figure S6. The cell uptake analysis of the AgZE nanoparticles for 24 h using ICPOES to analyze the uptake of AgZE both by the cancer (U-87) and normal cell (HEK-293) line.

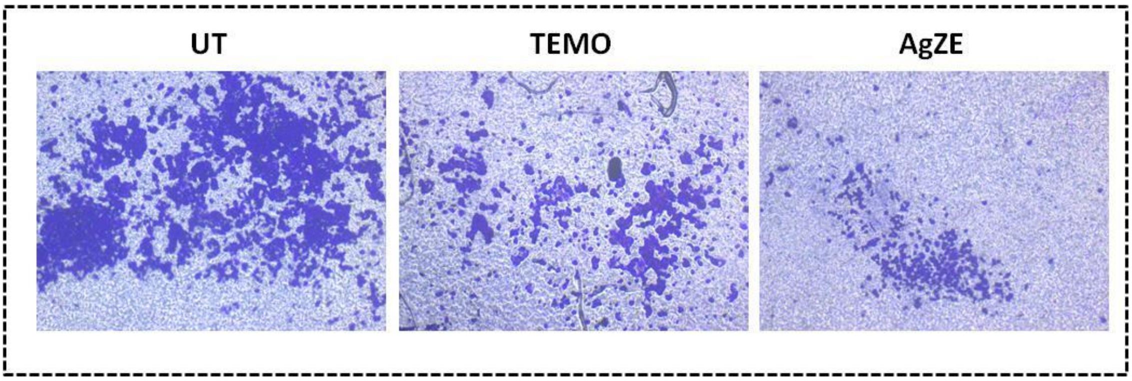

Figure S7. U-87 cell migration is observed for 14 h using transwell based migration assay.TheU-87 cell lines are incubated with i) untreated (first row), ii) temozolomide: TEMO, positive control (second row) and iii) AgZE (third row). The AgZE inhibits the migration of cells compared to untreated cells.

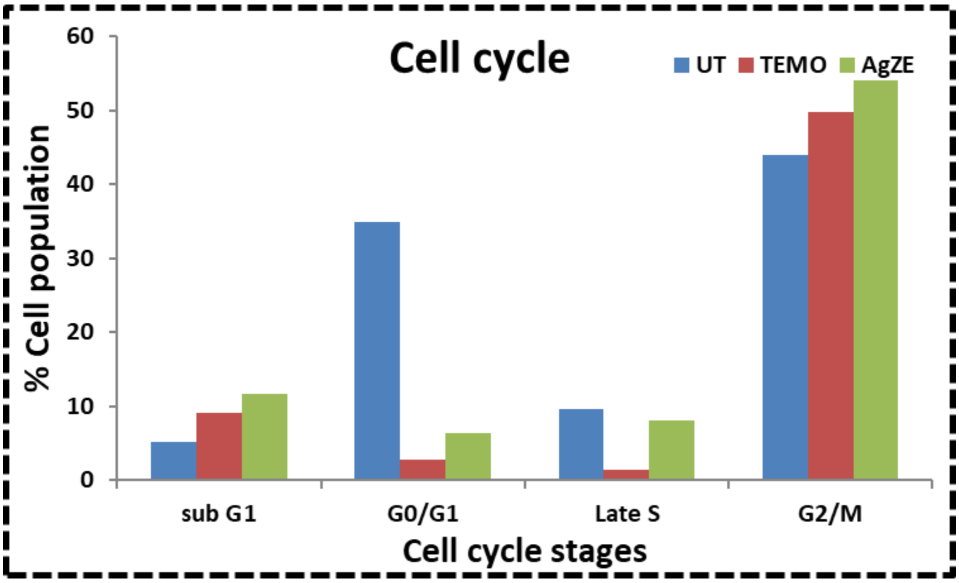

Figure S8. The graphical representation of cell cycle phases using flow cytometry analysis of U-87 cells at 24 h exhibiting sub G1 and G2/M phase arrest by AgZE treatment as compared to the untreated. Temozolomide is used as positive control.

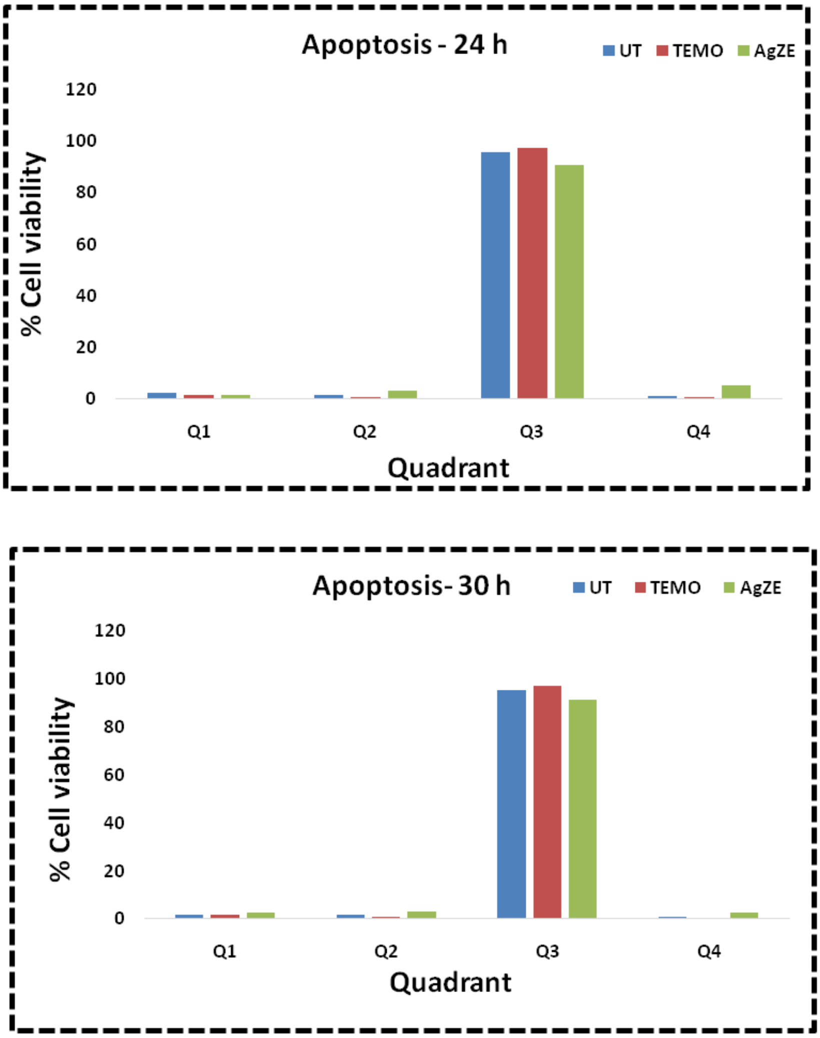

Figure S9. The graphical representation of apoptosis in the U-87 cell line treated with AgZE nanoparticles using Annexin V-FITC through flow cytometry analysis for 24 h and 30 h time points. Temozolomide is used as positive control.

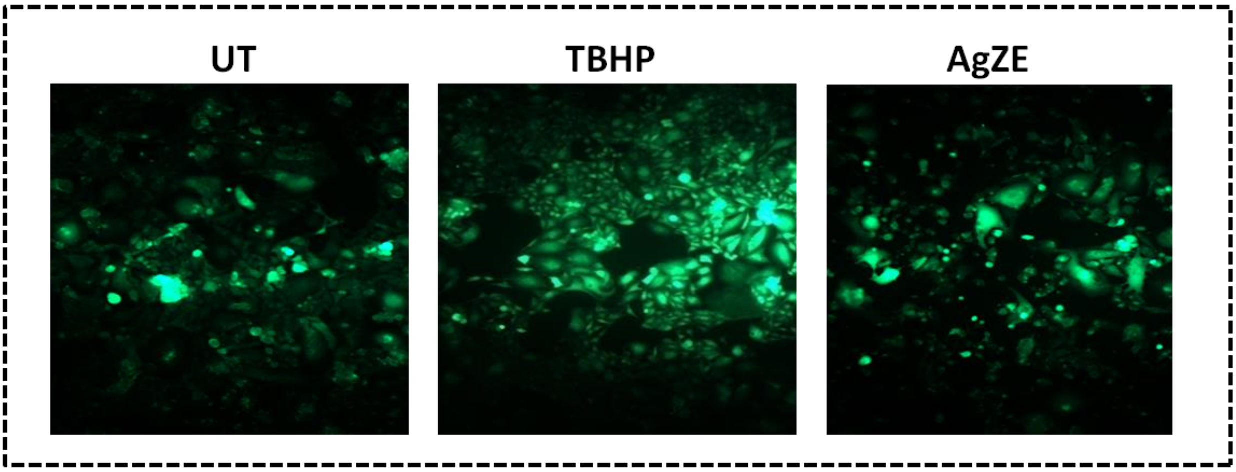

Figure S10. Determination of the intracellular ROS (H_2O_2) production in U-87 cell line using DCFDA reagent after treatment with AgZE (4.1µl) and TBHP (positive control) for 24 h. Higher amount of hydrogen peroxide production is observed in the U-87 cells upon treatment with AgZE compared to untreated (UT) cells.

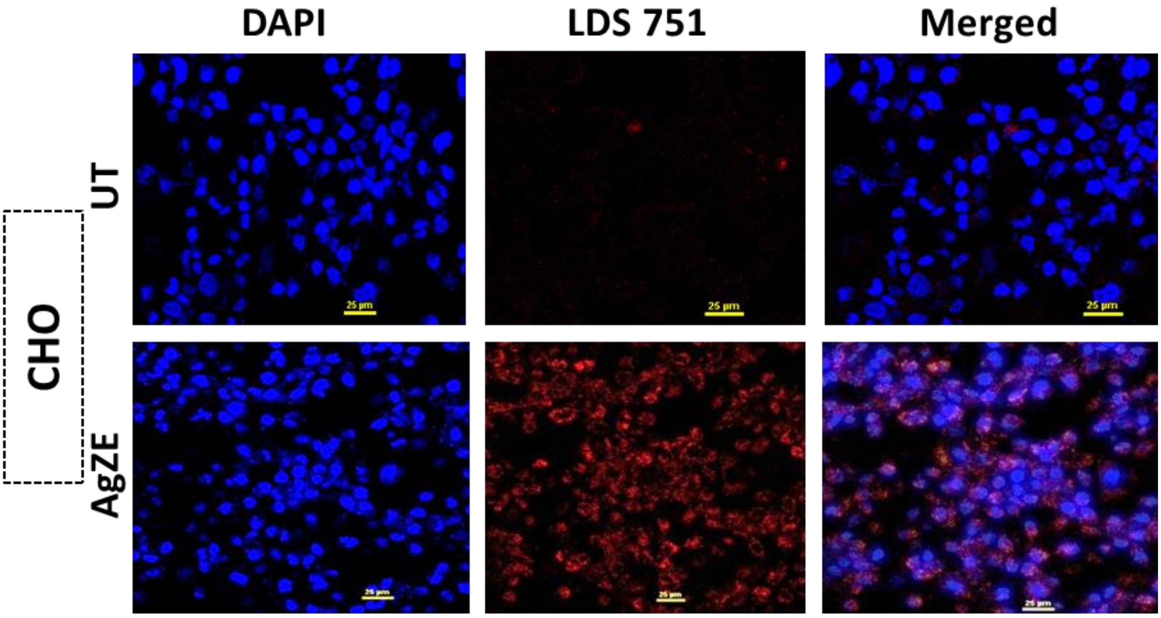

Figure S11. *In vitro* cellular uptake studies of AgZE in CHO cells using confocal microscopy (Row I: untreated; Row II: AgZE treated; Column I: Dapi; Column II: LDS 751; Column III: Merged). The red fluorescence from AgZE-treated cells is collected under the LDS 751 channel of the confocal microscope using a 60× objective.

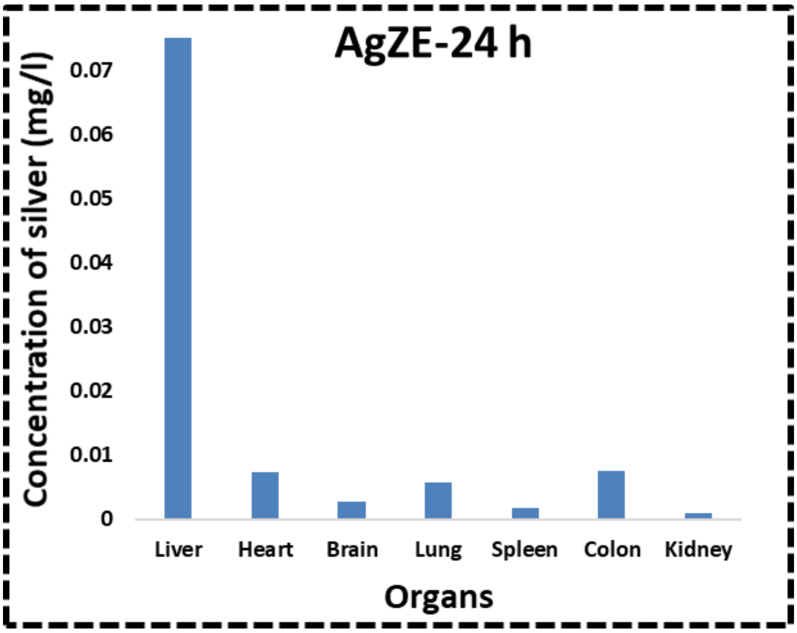

Figure S12. The ICPOES analysis of the C57BL6/J mice after intraperitoneal injection of AgZE to detect the presence of silver from AgZE in the liver, heart, brain, lung, spleen, colon and kidney.

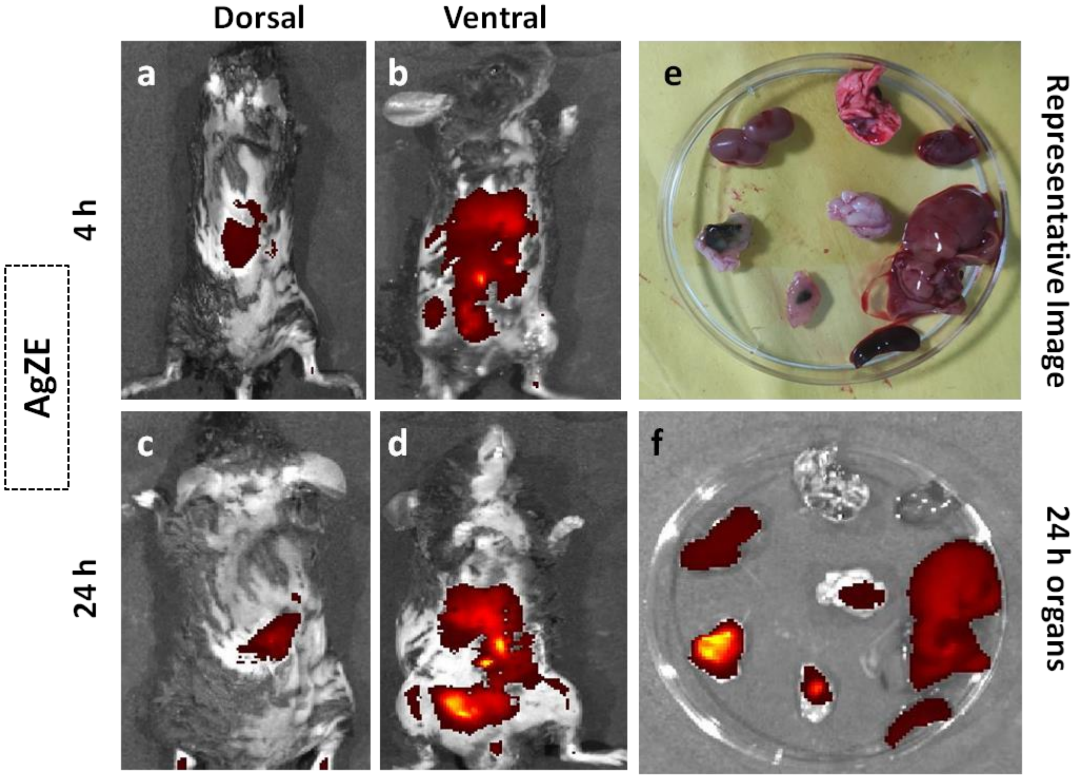

Figure S13. Pilot study of *In vivo* biodistribution of AgZE in melanoma tumor model in C57BL6/J mice at different time points, a) dorsal side at 4 h b) ventral side at 4 h c) dorsal side at 24 h d) ventral side at 24 h e) representative image of the tumor bearing mice organs at 24 h f) organs of mice at 24 h.

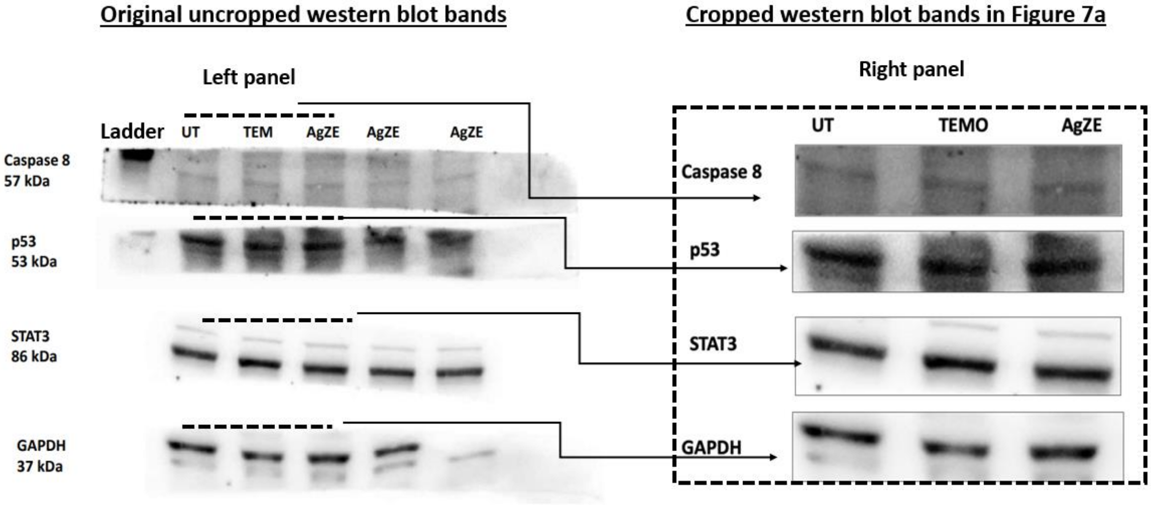

Figure S14. The left panel represents the original uncropped western blot bands from which the western blot bands on the right panel has been cropped for the manuscript file. Since we do not have cocktail antibody, therefore, we have cut the PVDF membrane into separate bands and incubated separately for each antibody with specific molecular weight as in Figure 7a.

References

1. Kotcherlakota, R.; Nimushakavi, S.; Roy, A.; Yadavalli, H.C.; Mukherjee, S.; Haque, S.; Patra, C.R. Biosynthesized Gold Nanoparticles: In Vivo Study of Near-Infrared Fluorescence (NIR)-Based Bio-imaging and Cell Labeling Applications. *ACS Biomater. Sci. Eng*. **2019**, *5*, 5439–5452.

2. Das, S.; Roy, A.; Barui, A.K.; Alabbasi, M.M.A.; Kuncha, M.; Sistla, R.; Sreedhar, B.; Patra, C.R. Anti-angiogenic vanadium pentoxide nanoparticles for the treatment of melanoma and their in vivo toxicity study. *Nanoscale*. **2020**, *12*, 7604–7621.
3. Mukherjee, S.; Kotcherlakota, R.; Haque, S.; Das, S.; Nuthi, S.; Bhattacharya, D.; Madhusudana, K.; Chakravarty, S.; Sistla, R.; Patra, C.R. Silver Prussian Blue Analogue Nanoparticles: Rationally Designed Advanced Nanomedicine for Multifunctional Biomedical Applications. *ACS Biomater. Sci. Eng*. **2020**, *6*, 690–704.
4. Ghanbari, M. Z.; Rastegari, P. M.; Mohammadi, M.h.; Mansouri, K. Cancer cells change their glucose metabolism to overcome increased ROS: One step from cancer cell to cancer stem cell? *Biomed. Pharmacother*. **2019**, *112*, 108690.
5. Souza, C.; Mônico, D.A.; Tedesco, A.C. Implications of dichlorofluorescein photoinstability for detection of UVA-induced oxidative stress in fibroblasts and keratinocyte cells. *Photochem. Photobiol. Sci*. **2020**, *19*, 40–48.
